# Supplementary material for: Capillary-associated microglia regulate vascular structure and function through PANX1-P2RY12 coupling in mice
Source: Nat Commun. 2021 Sep 6;12:5289. doi: 10.1038/s41467-021-25590-8 (PMC8421455; doi:10.1038/s41467-021-25590-8)
Supplement: Supplementary file 1 — Supplementary Information [file 41467_2021_25590_MOESM1_ESM.pdf]

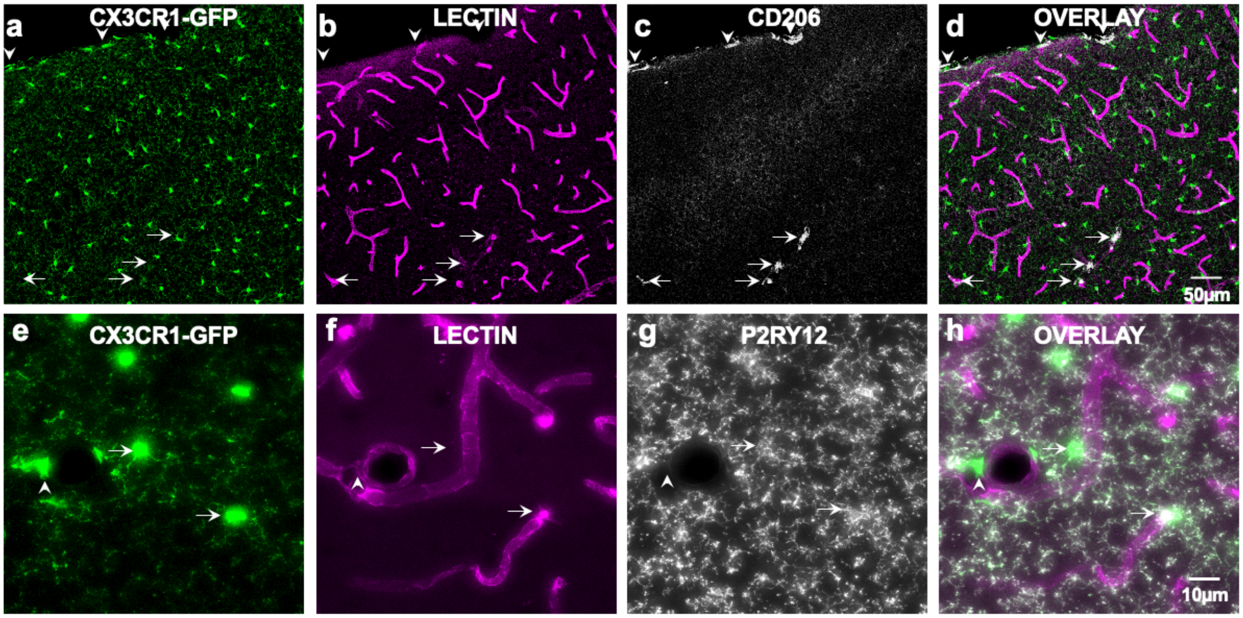

Supplementary Fig. 1: CD206 and P2RY12 molecular expression on ramified capillary-associated myeloid cells. a-d, Representative 20μm thick confocal projection images from a $CX3CR1^{GFP/+}$ adult brain showing myeloid cells (green), the vasculature (lectin in magenta) and $CD206^{+}$ macrophages (white arrows and arrowheads). $CD206^{+}$ cells are few in number but are localized either to the superficial meningeal layer (white arrowheads) or the vasculature in the cortex (white arrows). e-h, Representative 20μm thick projection images from a $CX3CR1^{GFP/+}$ adult brain showing myeloid cells (green), the vasculature (lectin in magenta) and $P2RY12^{+}$ microglia (white arrows) or a $P2RY12^{-}$ macrophage localized to a larger blood vessel (white arrowheads) in the cortex. Representative images were observed in 3 mice.

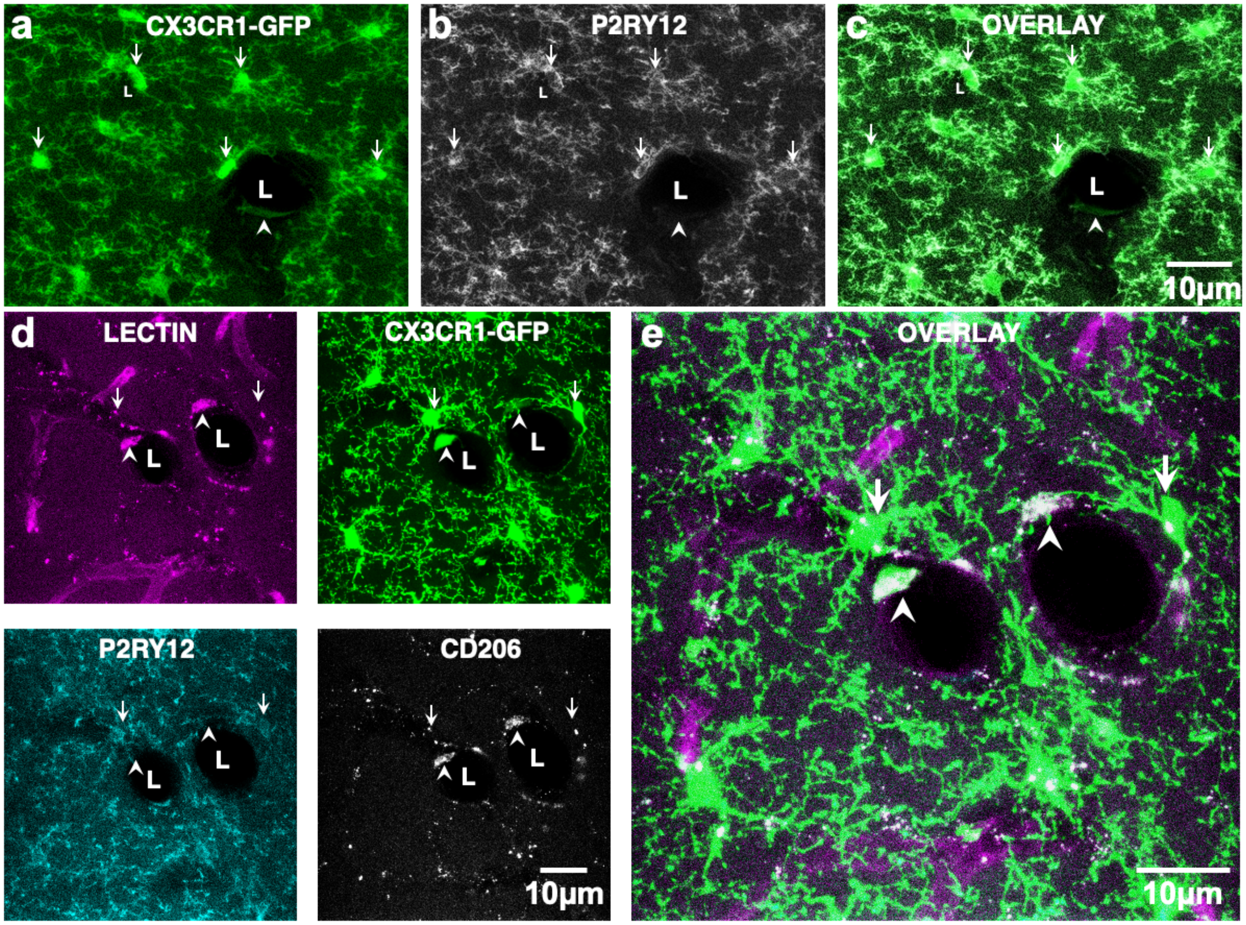

Supplementary Fig. 2: P2RY12 and CD206 expression on capillary-associated myeloid cells. a-c, Representative projection image from a CX3CR1$^{GFP/+}$ adult brain showing ramified myeloid cells (green) and P2RY12 expression (white). L identifies the lumen of a blood vessel, arrows, identify P2RY12^{+} microglia and arrowhead identifies a P2RY12^{-} myeloid cell likely to be a perivascular macrophage. d-e, Representative images of lectin-labelled (magenta) CX3CR1-expressing ramified cells (green), with P2RY12 expression (cyan) and CD206 (gray). Capillary-associated microglia are identified with white arrows and perivascular macrophages are identified in white arrowheads in d-e. Representative images were observed in 3 mice.

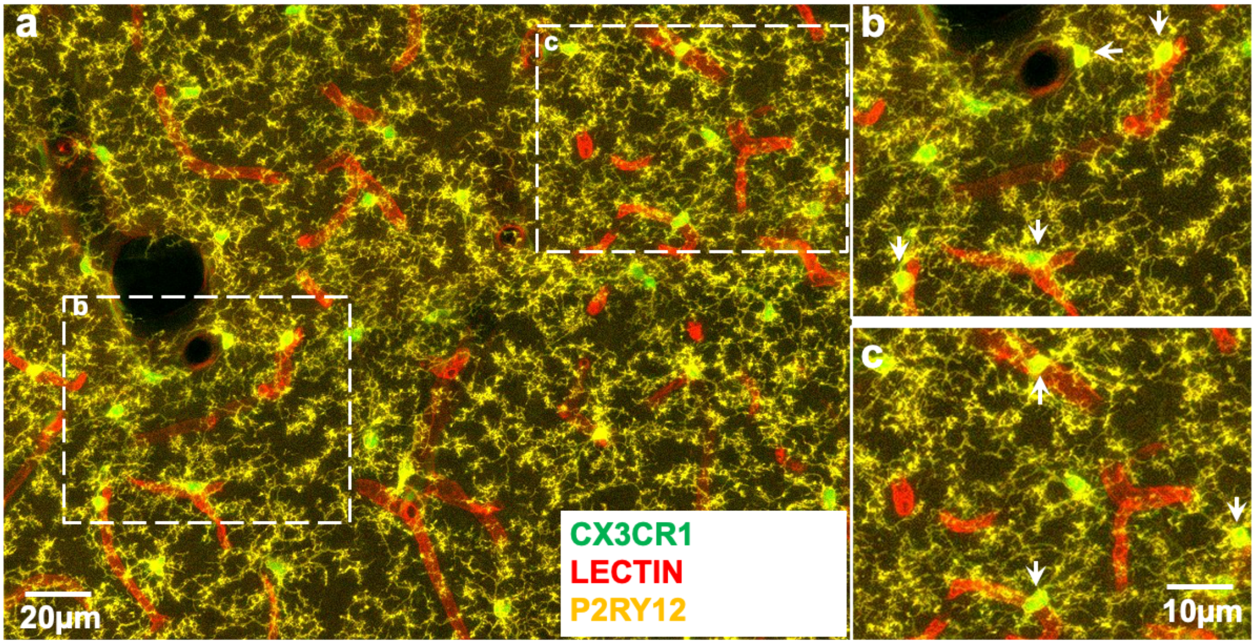

Supplementary Fig. 3: P2RY12 expression and capillary-associated myeloid cells. a-c, Representative images of CX3CR1-expressing ramified cells (green), lectin-labeled vasculature (red) and P2RY12 expression (yellow). Capillary-associated microglia are identified with white arrows in b-c. Representative images were observed in 3 mice.

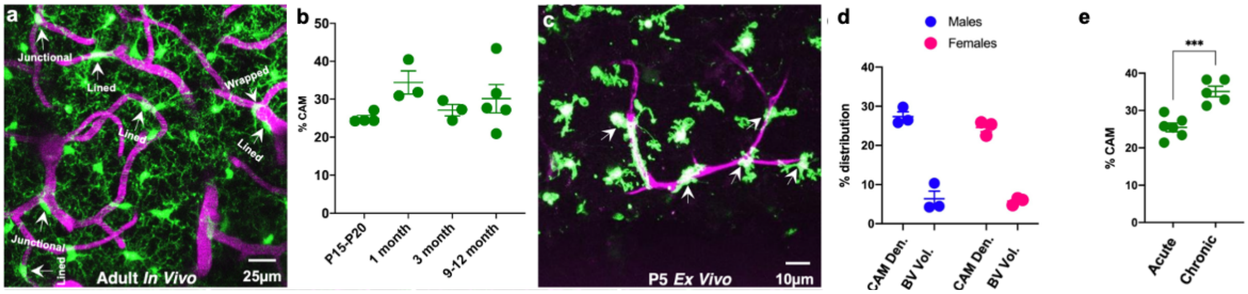

Supplementary Fig. 4: Characterization of capillary-associated microglia. a, Representative two photon *in vivo* projection image from a CX3CR1$^{GFP/+}$ adult brain showing various types of capillary-associated microglia (CAM) in green including "lined", "junctional" and "wrapped" CAMs with rhodamine-labelled blood vessels in magenta. b, Quantification of CAM density with age. c, Representative two photon image from a P5 brain slice showing CAMs (arrows) and microglia. (green) with lectin-labelled blood vessels (magenta). d, Distribution of CAM density relative to total microglia and blood vessel volume relative to whole brain volume in male and female mice. e, Quantification of CAM density through acutely and chronically implanted cranial windows. n = 3-5 fields of view from each of 3-6 mice in b, d and e. Representative images in a and c were observed in 3 mice. Data are presented as mean values ± SEM. ***p < 0.0001. Two-sided unpaired student T-test.

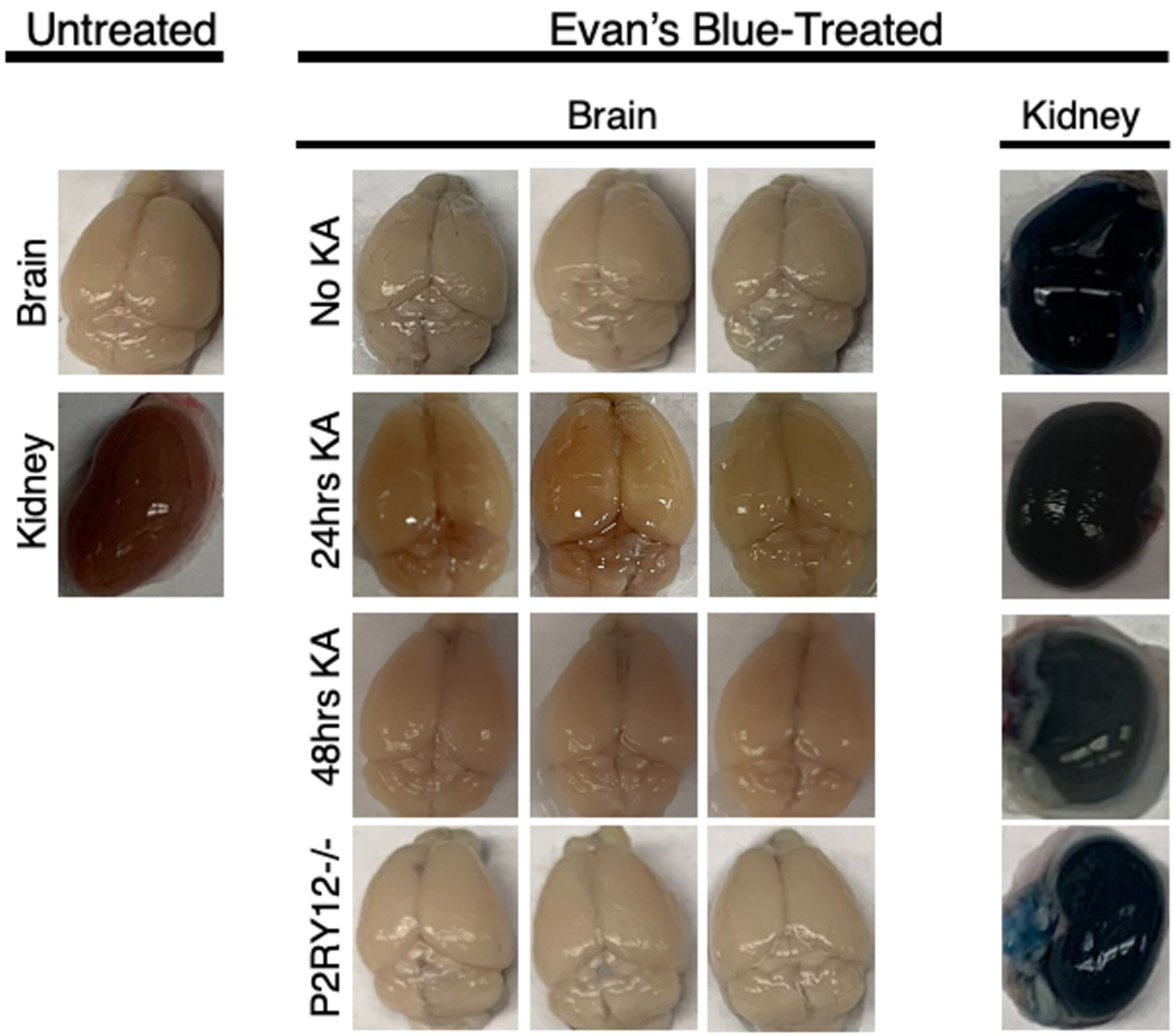

Supplementary Fig. 5: Assessment of blood brain barrier integrity. Mice in the various listed conditions were retro-orbitally treated with 2% Evan's Blue and euthanized 6 hours later. Mice brains and kidneys (as positive controls) were freshly excised, and pictures collected. Representative images were observed in 3 mice foreach condition.

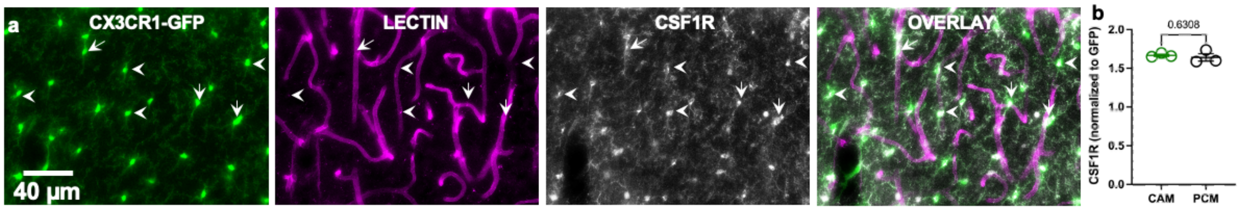

Supplementary Fig. 6: CSF1R expression in capillary-associated and parenchymal microglia. a, Representative images from a CX3CR1$^{GFP/+}$ adult brain showing microglia (green) capillaries (magenta) and CSF1R (gray) with capillary-associated microglia (CAM, arrows) parenchymal microglia (PCM, arrowheads). b, Quantification of microglial CSF1R expression in capillary-associated and parenchymal microglia. n = 3 mice each. Data are presented as mean values ± SEM. Two-sided unpaired student T-test.

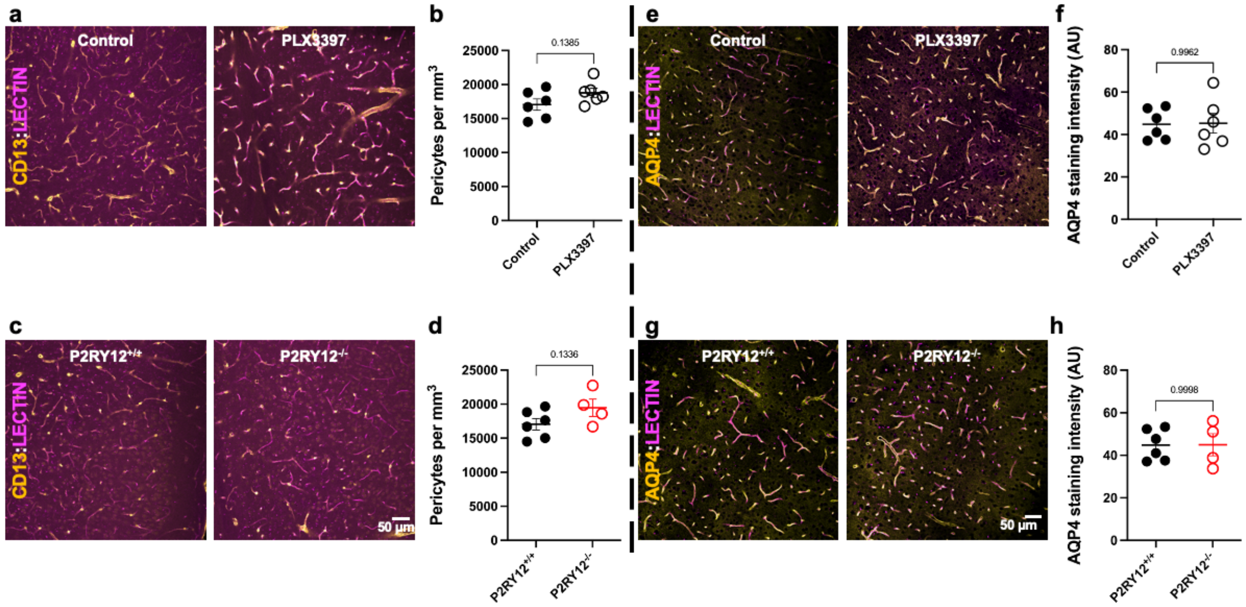

Supplementary Fig. 7: PLX3397 treatment or P2RY12 deficiency does not affect pericyte density or astrocytic endfeet coverage of the vasculature. a-d, Representative images (a, c) from control and PLX3397-treated (a) or P2RY12$^{+/+}$ and P2RY12$^{-/-}$ (c) mice as well as quantification of CD13 (yellow, pericyte) density (b, d) in control and PLX3397-treated (b) or P2RY12$^{+/+}$ and P2RY12$^{-/-}$ (d) mice with lectin-labelled blood vessels (magenta). e-h, Representative images (e, g) from control and PLX3397-treated (e) or P2RY12$^{+/+}$ and P2RY12$^{-/-}$ (g) mice as well as quantification of AQP4 (yellow, astrocytic endfeet) density (f, h) in control and PLX3397-treated (f) or P2RY12$^{+/+}$ and P2RY12$^{-/-}$ (h) mice with lectin-labelled blood vessels (magenta). n = 3 mice each. Data are presented as mean values ± SEM. Two-sided unpaired student T-test.

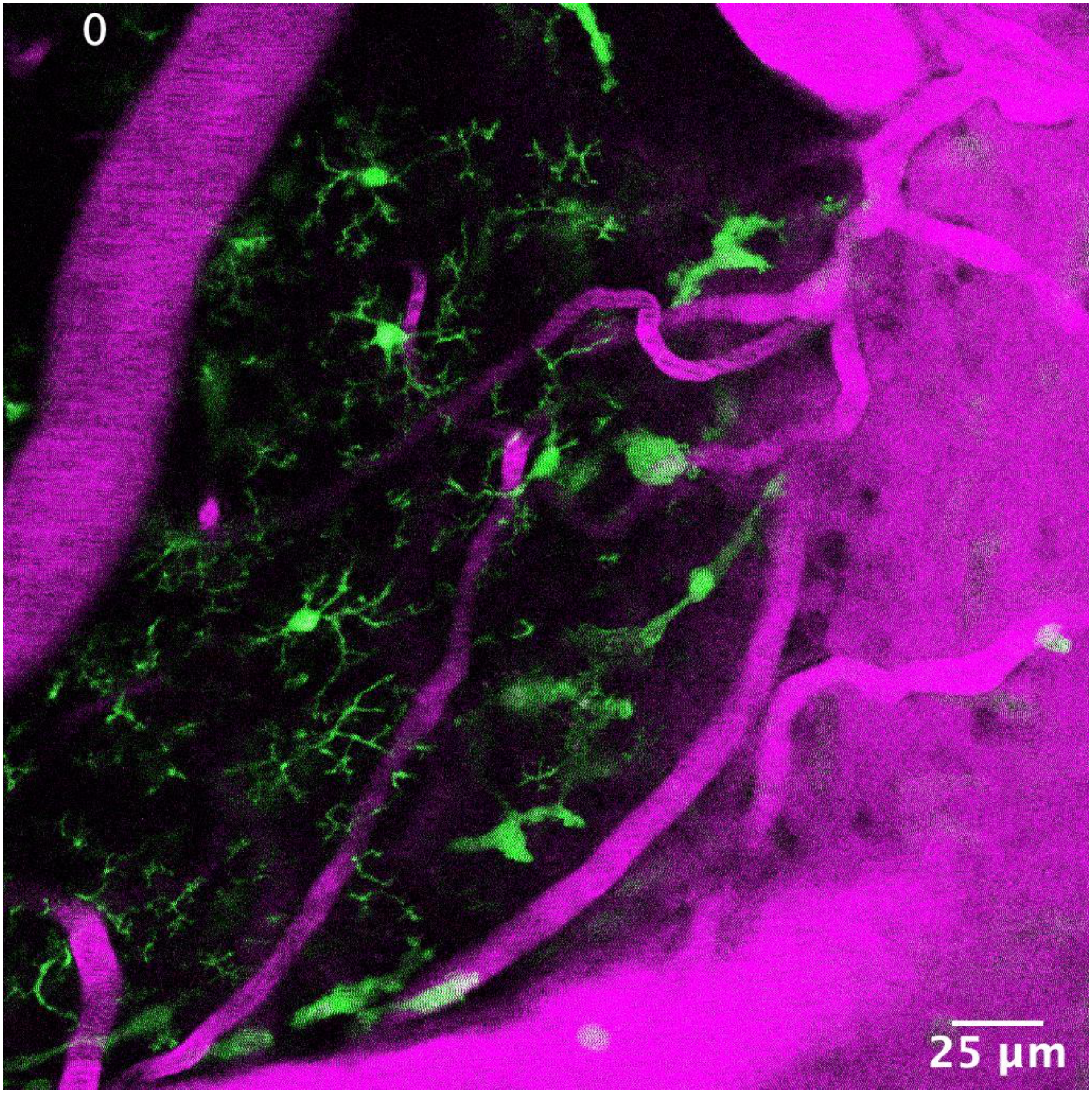

Supplementary Video 1: Ramified CX3CR1⁺ myeloid cells associate with brain capillaries *in vivo*. Representative *in vivo* thick two photon movie collected from a $CX3CR1^{GFP/+}$ adult brain showing myeloid cells (green) and the vasculature (rhodamine in magenta) at varying tissue depths between the brain surface and 200μm of the cortex. Arrows identify capillary-associated ramified myeloid cells.

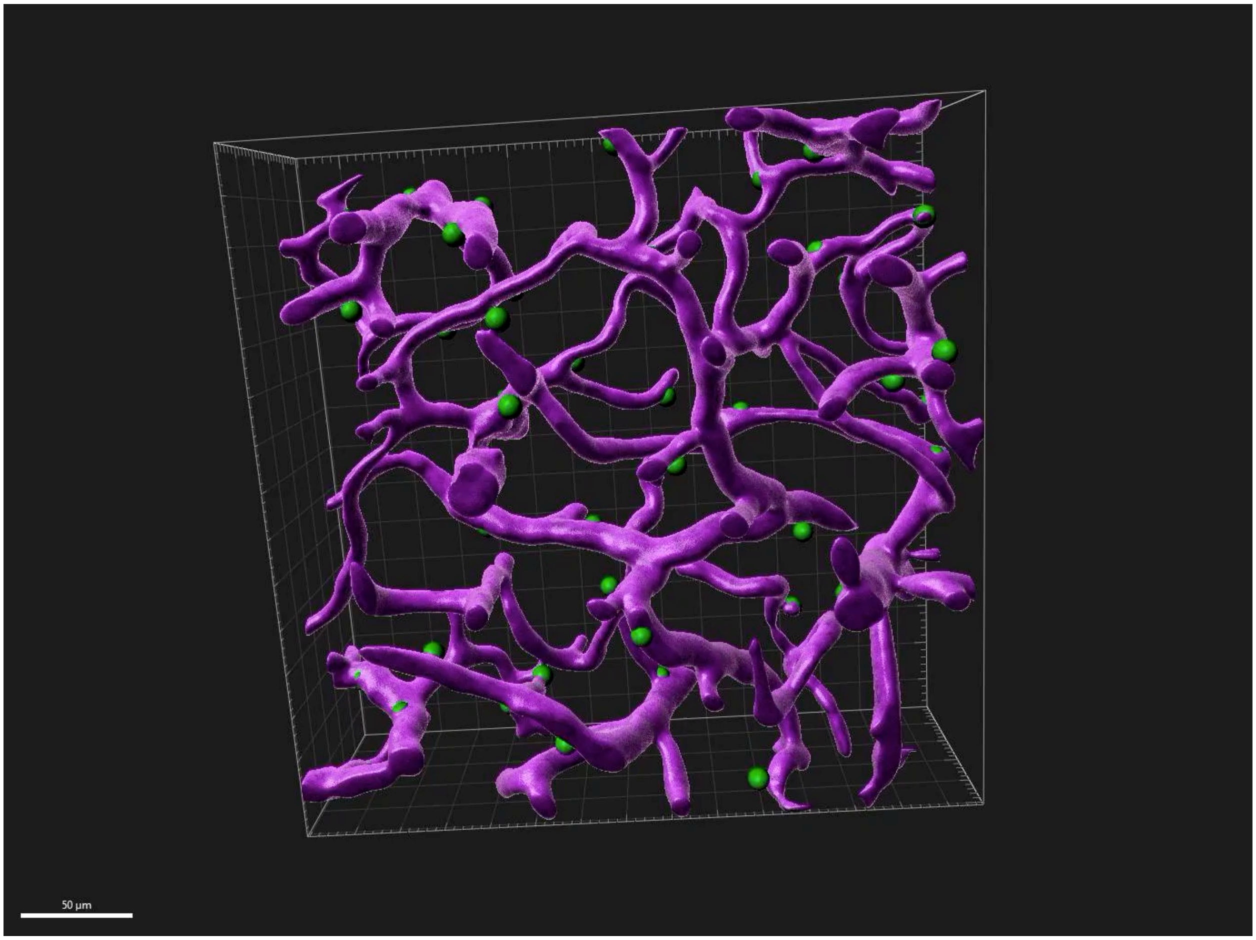

Supplementary Video 2: CX3CR1⁺ myeloid cell bodies associate with the vasculature *in vivo*. Representative 3D-reconstructed IMARIS movie collected from a CX3CR1$^{GFP/+}$ adult brain showing myeloid cell soma (green) on the vasculature (rhodamine in magenta).

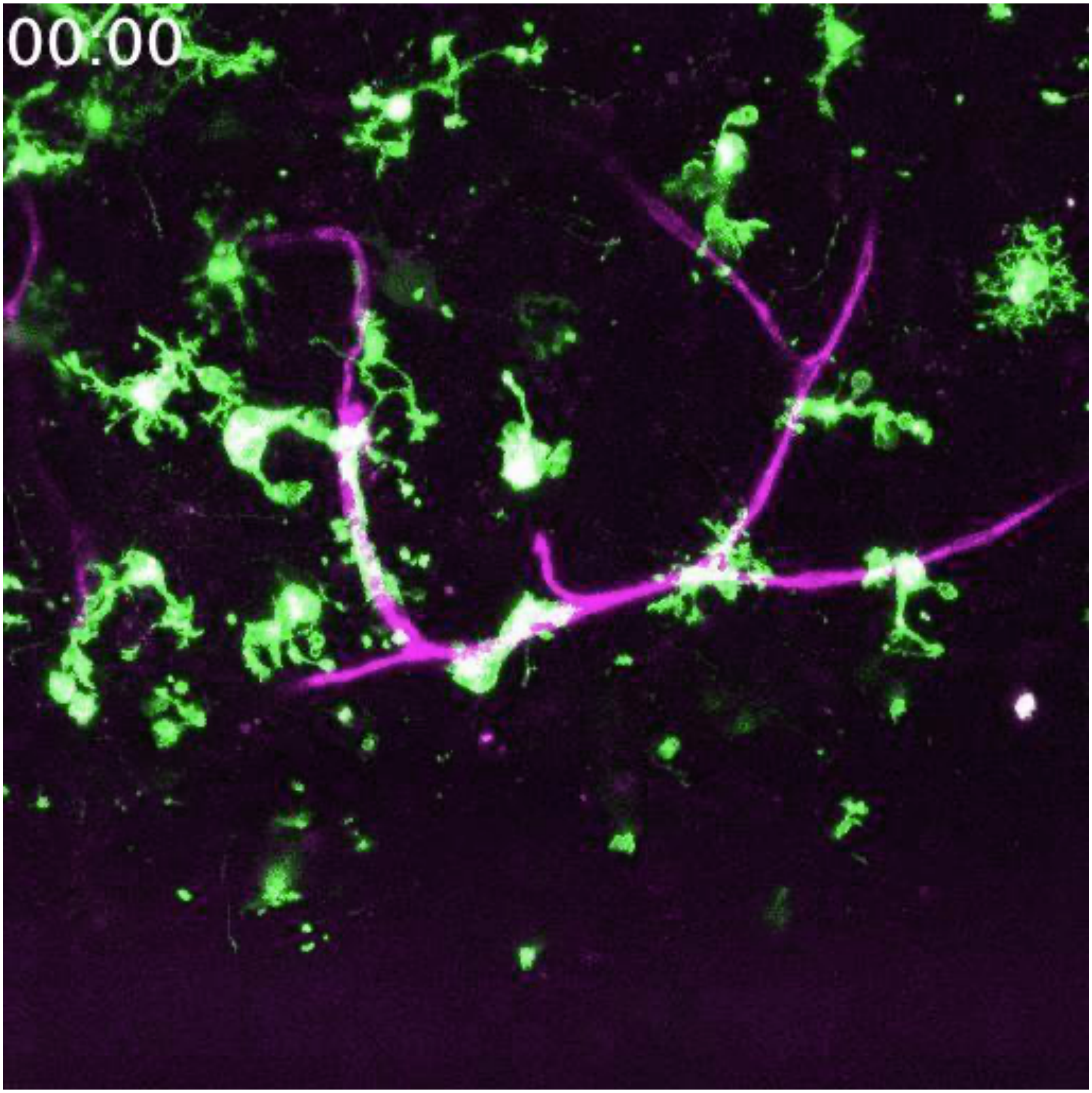

Supplementary Video 3: **Real-time imaging of neonatal capillary-associated microglia.** Representative movie collected from a P5 CX3CR1$^{GFP/+}$ mouse brain slice showing microglia (green) on the vasculature (magenta).

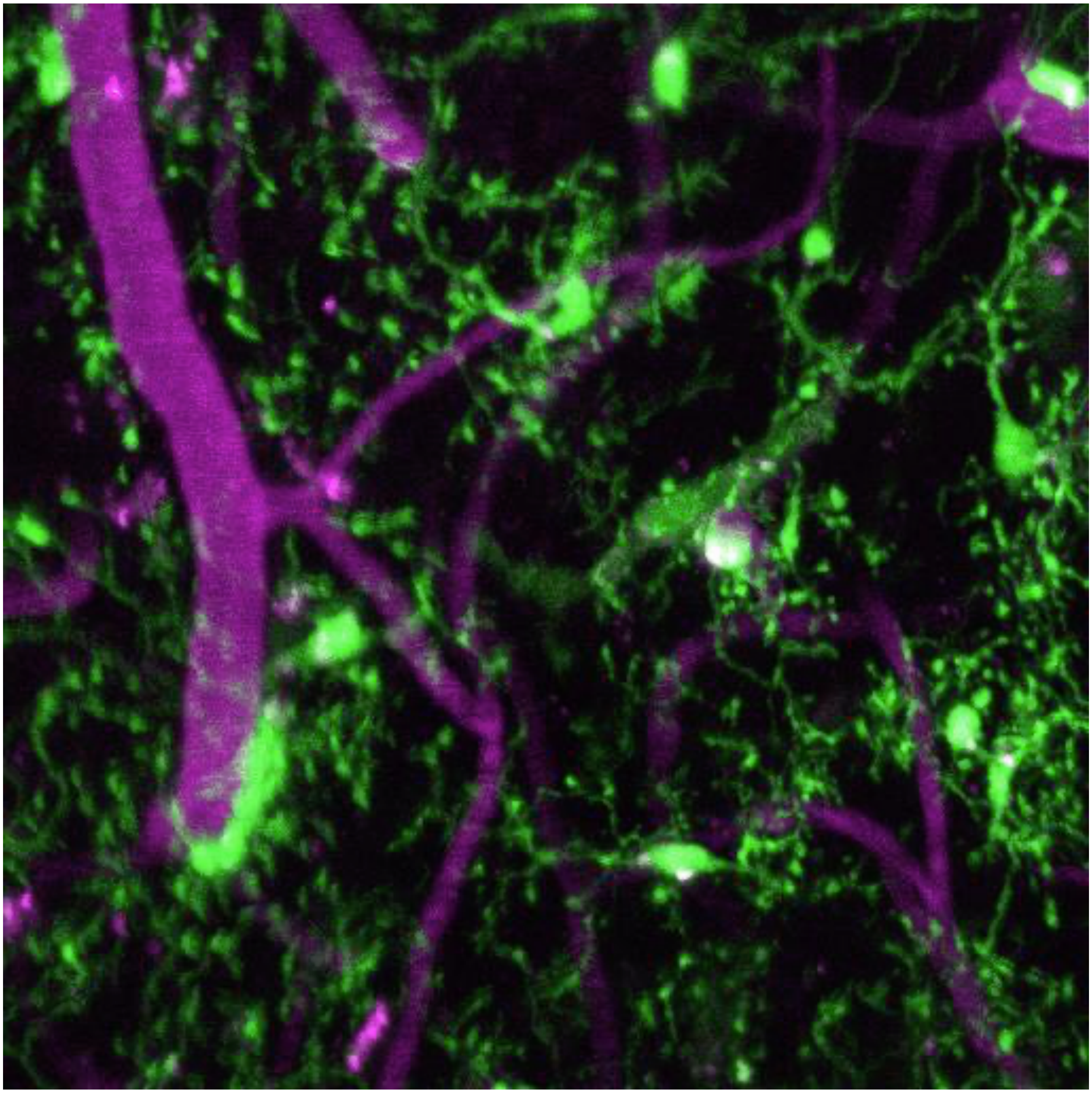

Supplementary Video 4: **Real-time imaging of capillary-associated and parenchymal microglia.** Representative movie collected from an adult $CX3CR1^{GFP/+}$ mouse *in vivo* showing capillary (magenta)-associated and parenchymal microglia (green) following a laser-induced injury.
