# Supplementary material for: Capillary-associated microglia regulate vascular structure and function through PANX1-P2RY12 coupling in mice
Source: Nat Commun. 2021 Sep 6;12:5289. doi: 10.1038/s41467-021-25590-8 (PMC8421455; doi:10.1038/s41467-021-25590-8)
Supplement: Supplementary file 3 — Description of Additional Supplementary Files [file 41467_2021_25590_MOESM3_ESM.docx]

Description of Additional Supplementary Files

Title: Supplementary Video 1

Description: Ramified CX3CR1+ myeloid cells associate with brain capillaries in vivo. Representative in vivo thick two photon movie collected from a CX3CR1GFP/+ adult brain showing myeloid cells (green) and the vasculature (rhodamine in magenta) at varying tissue depths between the brain surface and 200µm of the cortex. Arrows identify capillary-associated ramified myeloid cells.

Title: Supplementary Video 2

Description: CX3CR1+ myeloid cell bodies associate with the vasculature in vivo. Representative 3D-reconstructed IMARIS movie collected from a CX3CR1GFP/+ adult brain showing myeloid cell soma (green) on the vasculature (rhodamine in magenta).

Title: Supplementary Video 3

Description: Real-time imaging of neonatal capillary-associated microglia. Representative movie collected from a P5 CX3CR1GFP/+ mouse brain slice showing microglia (green) on the vasculature (magenta).

Title: Supplementary Video 4

Description: Real-time imaging of capillary-associated and parenchymal microglia. Representative movie collected from an adult CX3CR1GFP/+ mouse in vivo showing capillary (magenta)-associated and parenchymal microglia (green) following a laser-induced injury.
