# Supplementary material for: Capillary-associated microglia regulate vascular structure and function through PANX1-P2RY12 coupling in mice
Source: Nat Commun. 2021 Sep 6;12:5289. doi: 10.1038/s41467-021-25590-8 (PMC8421455; doi:10.1038/s41467-021-25590-8)
Supplement: Supplementary file 8 — Reporting Summary [file 41467_2021_25590_MOESM8_ESM.pdf]

nature research

Corresponding author(s): Ukpong B. Eyo

Last updated by author(s): Aug 12, 2021

Reporting Summary

Nature Research wishes to improve the reproducibility of the work that we publish. This form provides structure for consistency and transparency in reporting. For further information on Nature Research policies, see our Editorial Policies and the Editorial Policy Checklist.

Statistics

For all statistical analyses, confirm that the following items are present in the figure legend, table legend, main text, or Methods section.

n/a	Confirmed	
x		The exact sample size (*n*) for each experimental group/condition, given as a discrete number and unit of measurement
	x	A statement on whether measurements were taken from distinct samples or whether the same sample was measured repeatedly
	x	The statistical test(s) used AND whether they are one- or two-sided<br>*Only common tests should be described solely by name; describe more complex techniques in the Methods section.*
x		A description of all covariates tested
	x	A description of any assumptions or corrections, such as tests of normality and adjustment for multiple comparisons
	x	A full description of the statistical parameters including central tendency (e.g. means) or other basic estimates (e.g. regression coefficient) AND variation (e.g. standard deviation) or associated estimates of uncertainty (e.g. confidence intervals)
	x	For null hypothesis testing, the test statistic (e.g. *F*, *t*, *r*) with confidence intervals, effect sizes, degrees of freedom and *P* value noted<br>*Give P values as exact values whenever suitable.*
x		For Bayesian analysis, information on the choice of priors and Markov chain Monte Carlo settings
x		For hierarchical and complex designs, identification of the appropriate level for tests and full reporting of outcomes
x		Estimates of effect sizes (e.g. Cohen's *d*, Pearson's *r*), indicating how they were calculated

Our web collection on statistics for biologists contains articles on many of the points above.

Software and code

Policy information about availability of computer code

Data collection	In vivo two photon imaging data was obtained with the Leica microscope and software on a Leica SP8 Multi-photon microscope using the Leica Application Suite X version 3.5.7.23225 software. Confocal images were collected with a Leica TCS SP8 confocal microscope using the Leica Application Suite X version 3.5.5.19976 software. Non-confocal florescence images were collected with a Keyence microscope. Electron microscopy images were collected using a ORCAHR digital camera (10 MP; Hamamatsu). Laser speckle imaging was conducted to measure cerebral blood flow using the MoorFLPI-2 imaging system (Moor Instruments).
Data analysis	Image analysis was done using ImageJ (version 1.53k) to identify cells with cell bodies in close physical contact with the vasculature from two-photon, confocal and non-confocal florescence images. For 3D-reconstructed images, the built-in surface rendering function in IMARIS (version 9.7) was used for vascular architecture of the tissue and the percent volume of the vasculature within the tissue volume calculated by the software. BioRender was used to make schematics. For cerebral blood flow (CBF), laser speckle images were used to measure CBF in both brain hemispheres for at least 5 minutes using 16-color bands of perfusion units, and the absolute CBF perfusion units was averaged across both hemispheres.

For manuscripts utilizing custom algorithms or software that are central to the research but not yet described in published literature, software must be made available to editors and reviewers. We strongly encourage code deposition in a community repository (e.g. GitHub). See the Nature Research guidelines for submitting code & software for further information.

Data

Policy information about availability of data

All manuscripts must include a data availability statement. This statement should provide the following information, where applicable:

- Accession codes, unique identifiers, or web links for publicly available datasets
- A list of figures that have associated raw data
- A description of any restrictions on data availability

The data used to generate Fig. 1-7, Supplementary Fig. 1-7 and Supplementary videos 1-4 are available upon request.

Field-specific reporting

Please select the one below that is the best fit for your research. If you are not sure, read the appropriate sections before making your selection.

[x] Life sciences [ ] Behavioural & social sciences [ ] Ecological, evolutionary & environmental sciences

For a reference copy of the document with all sections, see nature.com/documents/nr-reporting-summary-flat.pdf

Life sciences study design

All studies must disclose on these points even when the disclosure is negative.

Sample size	No statistical methods were used to determine the required sample sizes before the experiments were conducted. Wee estimated using at least 3 mice per group and sometimes up to 8 mice per group and compared them by relevant statistics.
Data exclusions	No data was excluded
Replication	All experiments were repeated at least 3 times i.e. at least three mice were used. Data was collected by two to three experimenters. Data were analyzed by two and sometimes three experimenters and they were often blind. Different sets of experimenters analyzed different pieces of the data.
Randomization	Mice were randomly assigned to different experimental groups.
Blinding	The data analysis was often performed in a blinded manner with the analyzer being a different individual from the experimenter.

Reporting for specific materials, systems and methods

We require information from authors about some types of materials, experimental systems and methods used in many studies. Here, indicate whether each material, system or method listed is relevant to your study. If you are not sure if a list item applies to your research, read the appropriate section before selecting a response.

Materials & experimental systems

n/a	Involved in the study	
[ ]	[x]	Antibodies
[x]	[ ]	Eukaryotic cell lines
[x]	[ ]	Palaeontology and archaeology
[ ]	[x]	Animals and other organisms
[x]	[ ]	Human research participants
[x]	[ ]	Clinical data
[x]	[ ]	Dual use research of concern

Methods

n/a	Involved in the study	
[x]	[ ]	ChIP-seq
[x]	[ ]	Flow cytometry
[x]	[ ]	MRI-based neuroimaging

Antibodies

Antibodies used	Anti-P2Y12R (commercial), rabbit poly-clonal antibody from Ana Spec Cat. #: AS- 55043A verified in PMID: 29275859;<br>Anti-Iba1 (commercial), rabbit poly-clonal antibody from Wako. Catalog. #: 019-19741 verified in PMID: 29275859;<br>Anti-CD206 (commercial), rat monoclonal antibody from Biolegend, Catalog.# 141701 verified in PMID: 25991856;<br>Anti-CD31 (commercial), hamster monoclonal antibody from Millipore Catalog. # MAB1398Z verified in PMID: 31235908<br>Anti-CD13 (commercial), goat poly-clonal antibody from R&D Systems Catalog. # AF2335 verified in PMID: 31235908;<br>Anti-AQP4 (commercial), rabbit poly-clonal antibody from Sigma Aldrich Catalog.# A5971 verified in PMID: 24943270;<br>Anti-CSF1R (commercial), rabbit monoclonal antibody from Abcam Catalog.# ab254357 has no previous evidence of being verified.
Validation	All antibodies used are from commercial sources and have been previously validated my other studies. We validated them in our studies as well showing that they were expressed on cell types as expected.

Animals and other organisms

Policy information about studies involving animals; ARRIVE guidelines recommended for reporting animal research

Laboratory animals	Mice: 1. C57BL/6J (wildtype) mice; 2. B6.129P2(Cg)-Cx3cr1tm1Litt/J (CX3CR1-GFP) mice; 3. B6;FVB-Tg(Aldh1l1-EGFP/Rpl10a) JD130Htz/J (ALDH1L1-GFP) mice; 4. P2RY12-/- mice (not commercially available); and 5. PANX1-/- (not commercially available).
Wild animals	None
Field-collected samples	None
Ethics oversight	Institutional Animal Care and Use Committee at the University of Virginia

Note that full information on the approval of the study protocol must also be provided in the manuscript.
